# Temporal mismatch between pain behaviour, skin Nerve Growth Factor and intra-epidermal nerve fibre density in trigeminal neuropathic pain

**DOI:** 10.1186/1471-2202-15-1

**Published:** 2014-01-01

**Authors:** Laura J Evans, Alison R Loescher, Fiona M Boissonade, Simon A Whawell, Peter P Robinson, David Andrew

**Affiliations:** 1 Oral & Maxillofacial Medicine and Surgery, University of Sheffield School of Clinical Dentistry, Claremont Crescent, Sheffield, UK; 2 Oral & Maxillofacial Pathology, University of Sheffield School of Clinical Dentistry, Claremont Crescent, Sheffield, UK

**Keywords:** Trigeminal, Neuropathic pain, ELISA, hyperalgesia, TrkA

## Abstract

**Background:**

The neurotrophin Nerve Growth factor (NGF) is known to influence the phenotype of mature nociceptors, for example by altering synthesis of neuropeptides, and changes in NGF levels have been implicated in the pathophysiology of chronic pain conditions such as neuropathic pain. We have tested the hypothesis that after partial nerve injury, NGF accumulates within the skin and causes ‘pro-nociceptive’ phenotypic changes in the remaining population of sensory nerve fibres, which could underpin the development of neuropathic pain.

**Results:**

Eleven days after chronic constriction injury of the rat mental nerve the intra-epidermal nerve fibre density of the chin skin from had reduced from 11.6 ± 4.9 fibres/mm to 1.0 ± 0.4 fibres/mm; this slowly recovered to 2.4 ± 2.0 fibres/mm on day 14 and 4.0 ± 0.8 fibres/mm on day 21. Cold hyperalgesia in the ipsilateral lower lip was detectable 11 days after chronic constriction injury, although at this time skin [NGF] did not differ between sides. At 14 days post-injury, there was a significantly greater [NGF] ipsilaterally compared to contralaterally (ipsilateral = 111 ± 23 pg/mg, contralateral = 69 ± 13 pg/mg), but there was no behavioural evidence of neuropathic pain at this time-point. By 21 days post-injury, skin [NGF] was elevated bilaterally and there was a significant increase in the proportion of TrkA-positive (the high-affinity NGF receptor) intra-epidermal nerve fibres that were immunolabelled for the neuropeptide Calcitonin Gene-related peptide.

**Conclusions:**

The temporal mismatch in behaviour, skin [NGF] and phenotypic changes in sensory nerve fibres indicate that increased [NGF] does not cause hyperalgesia after partial mental nerve injury, although it may contribute to the altered neurochemistry of cutaneous nerve fibres.

## Background

The neurotrophin Nerve Growth Factor (NGF) is essential for normal development of the embryonic nervous system [1], and in adult animals it maintains the phenotype of Aδ- and C-fibre nociceptors as well as sympathetic efferents [2-6]. NGF is produced in the target tissues that are innervated by NGF-dependent nerve fibres; in the skin for example, the major source of NGF is the proliferating layer of keratinocytes in the basal epidermis [7,8] but fibroblasts, Merkel cells and skeletal muscle also synthesize NGF [9,10]. Intradermal and intramuscular injections of NGF cause persistent heat and mechanical hyperalgesia in humans [11-14] and mutations in the high affinity NGF receptor TrkA (Tropomyosin-related kinase A) are associated with the rare condition congenital pain insensitivity with anhidrosis [15].

NGF causes pain by temporally-distinct pathophysiological mechanisms, which have mostly been identified in inflammatory models of persistent pain [16,17]. Initially, NGF directly sensitizes nociceptors [18], probably the neuropeptide-containing C-fibre and Aδ nociceptors, as the majority of them express TrkA receptors [19]. This is followed by later changes in protein transcription in the cell bodies of nerve fibres due to retrograde transport of NGF. There is increased synthesis of peptides (*e.g.* substance P, Calcitonin gene-related peptide; [20]) and ion channels [21], and indirect effects on synaptic transmission in the spinal cord have also been described [22].

As well as having a role in the pathophysiology of inflammatory pain, NGF has also been proposed to be important in neuropathic pain [23]. Systemic administration of NGF neutralizing antibodies has anti-hyperalgesic and anti-allodynic effects in several models of neuropathic pain [24-26], although whether the effects of NGF neutralization occur centrally or peripherally is not known. One hypothesis is that excessive NGF in the periphery coupled with a reduced innervation density, which occurs in virtually all neuropathic pains with a peripheral origin, causes nociceptor sensitization via phenotypic changes, and ultimately chronic pain [23]. There is some support for this hypothesis in painful diabetic neuropathy [27], where skin NGF levels are increased and systemic NGF antibodies reversed behavioural signs of neuropathic pain, but it is not known if this mechanism is specific to diabetic neuropathy. Here we have investigated the relationship between NGF changes in the skin after partial nerve injury, the phenotype of intra-epidermal nerve fibres and how these relate to behavioural signs of neuropathic pain. We show that skin NGF levels are unchanged when there is neuropathic pain, and phenotypic alterations in skin nerve fibres are probably unrelated to NGF levels.

## Results

### Neuropathic pain after partial injury to the mental nerve

Two animals were excluded from the experiment as they could not be trained to feed from the experimenter’s fingers. Two other rats were excluded as they consistently displayed aversive responses to the weakest von Frey hair pre-operatively, and it would have been impossible to identify mechanical hyperalgesia/allodynia post-surgery. Dominant rats were trained the most rapidly. In one group of rats the most submissive individual would only perform the test with another rat in the cage. In this case the next most submissive rat was allowed in the cage during testing which proceeded with minimal interference.

**Figure 1:**
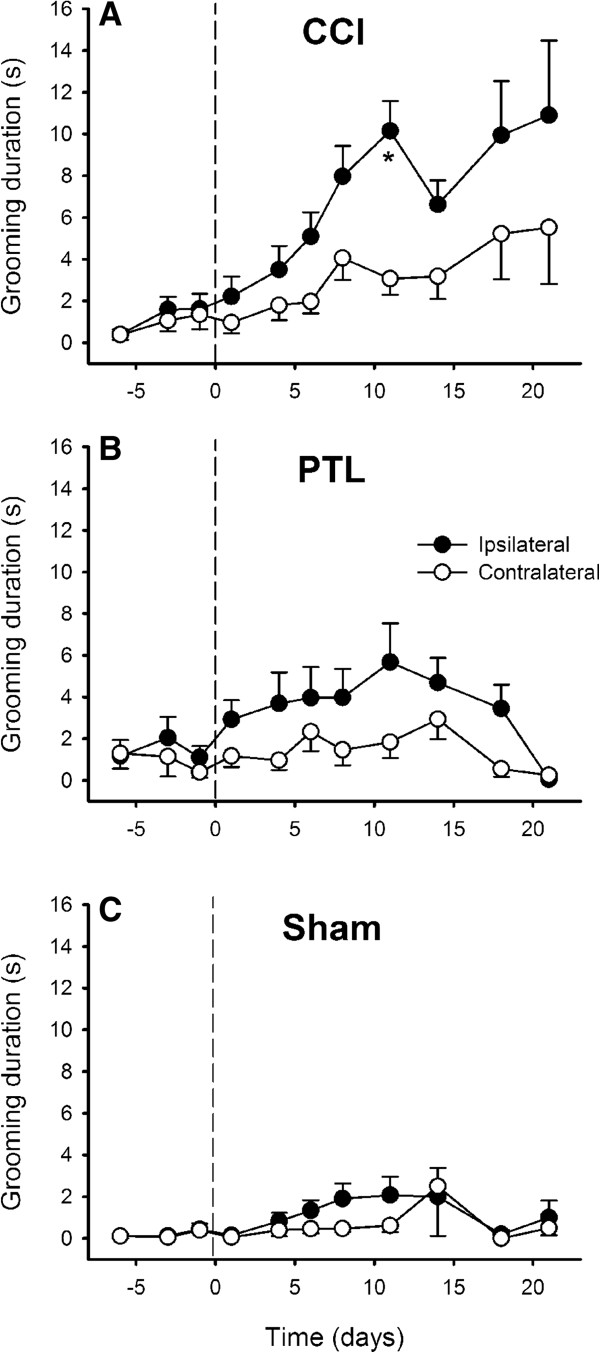
**Time course of the development of cold-evoked behaviours after mental nerve injury.** Bilateral grooming evoked by cold stimulation (topical ethyl chloride) of the chin was significantly increased 11 days after chronic constriction injury (CCI) of a mental nerve **(A)**, but not after partial tight ligation of the nerve (PTL; **B**) or sham operation **(C)**. The operation was performed on day 0 (indicated by the dashed line). Symbols are mean ± 1 S.E.M. Asterisk indicates significant differences between ipsilateral and contralateral sides (*P* < 0.002; 2-factor repeated measures *ANOVA* followed by Tukey’s test *post-hoc*). CCI, *n* = 22; PTL, *n* = 19; Sham, *n* = 17.

There was usually very little or no grooming in response to cold stimuli in naïve rats. The typical aversive response to a cold stimulus was bilateral grooming of the chin. Other responses including fast withdrawal and wiping the stimulated area on the cage floor were observed occasionally and these behaviours seemed to be more common after the CCI, although they were not quantified. Cold-evoked bilateral grooming increased significantly after CCI (*P* < 0.001, RM *ANOVA*), and was greater ipsilaterally (Figure 1). When compared to the contralateral side, significantly more grooming was evoked 11 days after the CCI (*P* < 0.002, Tukey’s test; Figure 1). *P*-values for ispilateral grooming duration on days 14, 18 and 21 approached significance, but were not less than 0.05. Both sham-operated and animals with a PTL injury of the mental nerve showed no significant difference in grooming after cold stimultion (Figure 1). No grooming or other aversive reaction were ever evoked by application of a dry cotton bud (neutral temperature) indicting that there was no confounding factor of tactile allodynia in nerve injured animals (and confirming the data obtained with von Frey filaments, see following text).

**Figure 2:**
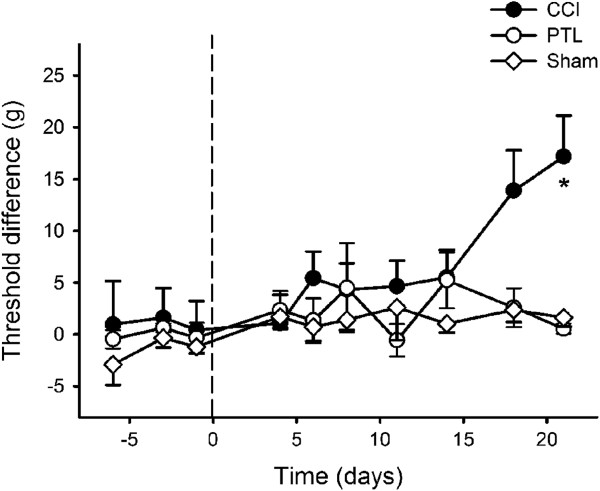
**Mechanical sensitivity after nerve injury.** 50% withdrawal threshold differences between operated and non-operated sides were significantly different 21 days after chronic constriction injury (CCI) of the mental nerve, but not after partial tight ligation (PTL) or sham operation. The operation was performed on day 0 (indicated by the dashed line). Symbols are mean ± 1 S.E.M. Asterisk indicates significant differences between ipsilateral and contralateral sides (*P* < 0.003; 2-factor repeated measures *ANOVA* followed by Tukey’s test *post-hoc*).

**Figure 3:**
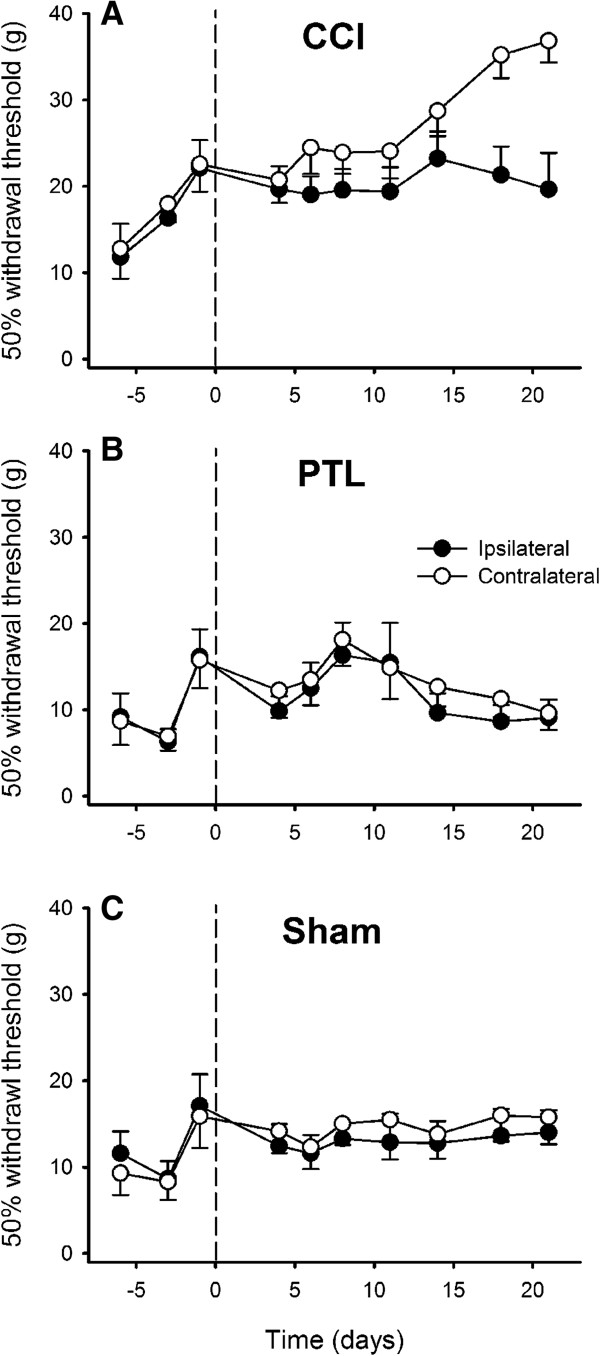
**Time course of the development of mechanically-evoked behaviours after injury of the mental nerve.** 50% withdrawal thresholds evoked by graded stimulation of the chin hairy skin with von Frey monofilaments after chronic constriction injury (CCI; **A**), partial tight ligation (PTL; **B**) and sham operation **(C)** of the mental nerve. The operation was performed on day 0 (indicated by the dashed line). Symbols are mean ± 1 S.E.M.

Mechanical stimulation of the chin with noxious-intensity von Frey filaments caused flinches or fast withdrawals from the stimulus. Snatching the food from the experimenter’s fingers during testing could be mistaken for an aversive response, and if this occurred the food was immediately taken away by the experimenter to train the animals not snatch. CCI (*P* < 0.001, General Linear model) but not PTL (*P* > 0.2, General Linear model) caused mechanical 50% withdrawal thresholds to change significantly after mental nerve injury (Figure 2): they were significantly higher on day 21 compared to threshold differences pre-injury (*P* < 0.03 Tukey’s *post-hoc* test). Maximum threshold difference between sides on day 21 was 16.4 g (ipsilateral = 15.9 ± 3.0 g [mean ± SEM]; contralateral = 32.3 ± 2.6 g), but this was due to hypoalgesia contralaterally (*P* < 0.003, *ANOVA*), rather than hyperalgesia ipsilaterally (Figure 3).

**Figure 4:**
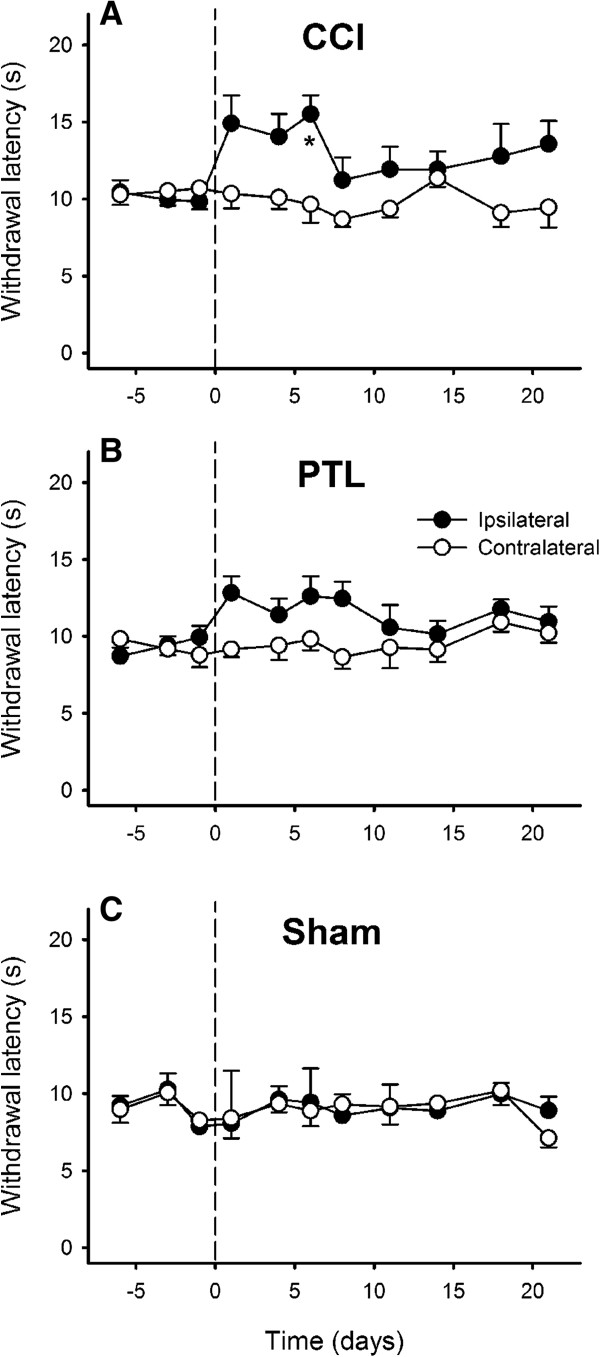
**Time course of the development of heat-evoked behaviours after injury of the mental nerve.** Withdrawal latencies to radiant heat stimulation of the chin were significantly increased 6 days after chronic constriction injury (CCI) of a mental nerve **(A)**, but not after partial tight ligation of the nerve (PTL; **B**) or sham operation **(C)**. The operation was performed on day 0 (indicated by the dashed line). Symbols are mean ± 1 S.E.M. Asterisk indicates significant differences between ipsilateral and contralateral sides (*P* < 0.03; 2-factor repeated measures *ANOVA* followed by Tukey’s test *post-hoc*).

Aversive responses to heat stimuli were fast withdrawals away from the radiant heat stimulus that had latencies of 7 – 12 s in unoperated rats. After CCI, there was a transient increase in heat-evoked withdrawal latency ipsilaterally (*P* < 0.03, RM *ANOVA*) at 6 days post-injury (*P* < 0.03, Tukey’s test), whereas contralateral withdrawal latencies remained stable, thus indicating temporary heat hypoaesthesia on the operated side. There was no significant change in withdrawl latency to heat stimuli after either PTL injury to the mental nerve or a sham-operation (Figure 4).

### Skin [NGF] in neuropathic pain after partial nerve injury

Because only animals with a CCI of the mental nerve showed behavioural signs of neuropathic pain, the PTL injury model was not studied further. Skin samples were obtained from animals that were killed on post-operative days 11, 14 and 21 as these time points showed behavioural evidence of neuropathic pain. To enrich the study, tissue from rats that showed the largest differences between cold-evoked grooming behaviour on post-operative day 11 were selected for assay (*n* = 6 per group).

**Figure 5:**
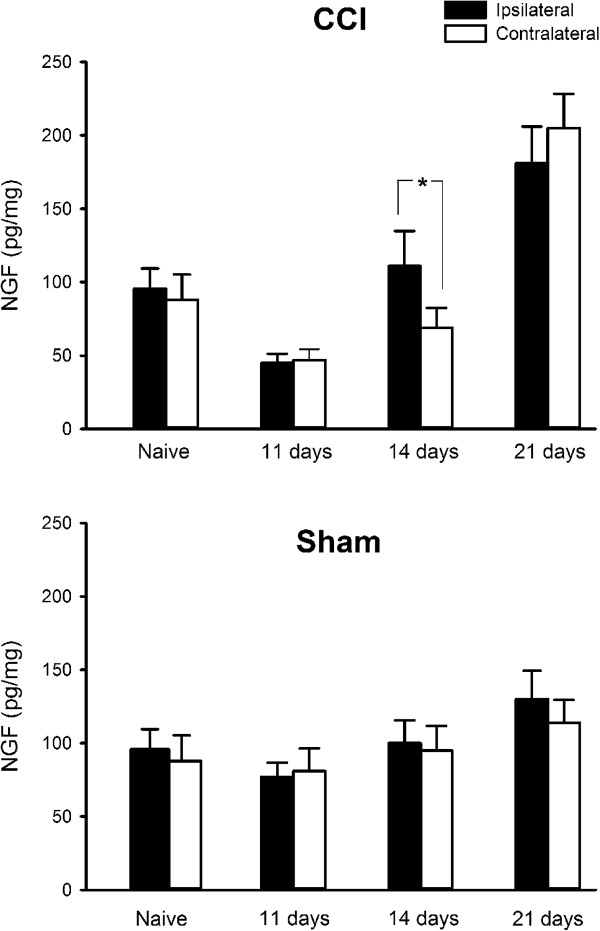
**Change in NGF concentration in rat lower lip skin after chronic constriction injury (CCI) or sham-operation.** Bars show skin NGF levels in naive rats and 11, 14 and 21 days after the operation. At 14 days post-injury [NGF] was significantly higher ipsilateral to a CCI compared to the contralateral side (*P* < 0.04, *n* = 6/group).

The average [NGF] in unoperated lip skin was 92 pg/mg (range: 76 – 115; S.D. 15.3, *n* = 10) and there was no significant difference between sides (Ipsilateral 96 ± 13.7 pg/mg, Contralateral 88 ± 17.4 pg/mg; *P* > 0.4, *t*-test). Eleven days after mental nerve CCI, when there was cold hyperalgesia behaviourally, skin [NGF] was significantly lower than in naïve and sham-operated controls (*P* < 0.01 *ANOVA* followed by Tukey’s test *post-hoc*, Figure 5) and this occurred bilaterally. Over time [NGF] slowly increased bilaterally (*P* < 0.002, *ANOVA*), so that by day 21 there was significantly more NGF in chin skin compared to naïve and sham-operated controls (*P* < 0.05, 2-factor *ANOVA* followed by Tukey’s test *post-hoc*). There was significantly more NGF ipsilaterally compared to contralaterally 14 days after a CCI, (*P* < 0.05, *ANOVA*; Figure 5; ipsilateral: 111 ± 23.9 pg/mg (mean ± SD), contralateral: 69 ± 13.4 pg/mg), but not after either 11 (*P* = 0.8) or 21 days (*P* = 0.5). Compared to sham-operated animals on day 14, [NGF] was not significantly different (*P* > 0.5, 2 factor *ANOVA*) in CCI rats. There was no significant change in NGF levels in sham operated animals over time (Figure 5; *P* > 0.09, ANOVA).

### Density and phenotype of intra-epidermal nerve fibres

The innervation density and neurochemical phenotype of intra-epidermal nerve fibres (IENFs) supplying the mental nerve territory were determined from samples obtained from the same animals that were used for assay of skin [NGF].

**Figure 6:**
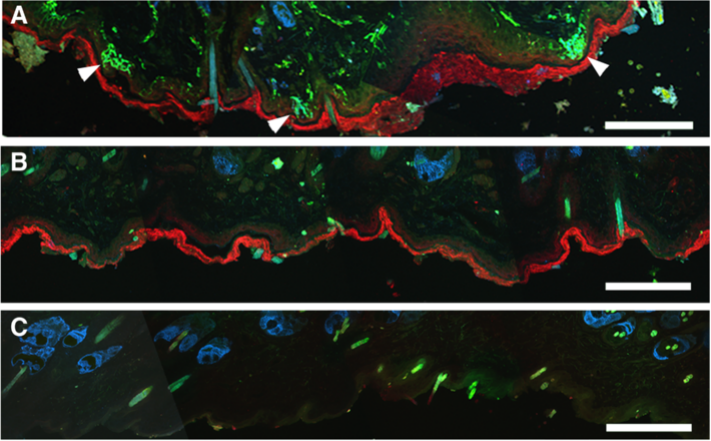
**Immunocytochemical labelling and controls.** Positive labelling **(A)** with antibodies to protein gene-product 9.5 (PGP 9.5, green), the high-affinity nerve growth factor receptor TrkA (Tropomyosin-related kinase A, blue) and the neuropeptide Calcitonin gene-related peptide (CGRP, red). Arrowheads indicate intra-epidermal nerve fibres. Pre-absorbing the primary antibodies with an excess of their immunogenic proteins abolished all nerve fibre labelling **(B)**, but the stratum corneum labelling persisted unless the CGRP antibody was omitted **(C)**, presumably due to cross-reactivity of the CGRP antibody with keratin. Dermal sebaceous glands were labelled in pre-absorption **(B)** and negative controls **(C)**, probably due to endogenous biotin. Hairs autofluoresced and were either FITC or coumarin-blue labelled. Scale bar = 100 μm.

No neuronal structures could be seen either after omission of the primary antibodies or preabsorption of the antibodies with their immunogens (Figure 6). The stratum corneum was Cy3-labelled in pre-absorption controls but not when the primary anti-CGRP antibody was omitted, this was presumably due to antibody cross-reactivity with keratin. Sebaceous glands in the dermis were labelled in both positive and negative control experiments, and this was attributed to endogenous biotin within the glands [28]. Hairs autofluoresced and were visible in either the FITC or coumarin channels in controls (Figure 6).

**Figure 7:**
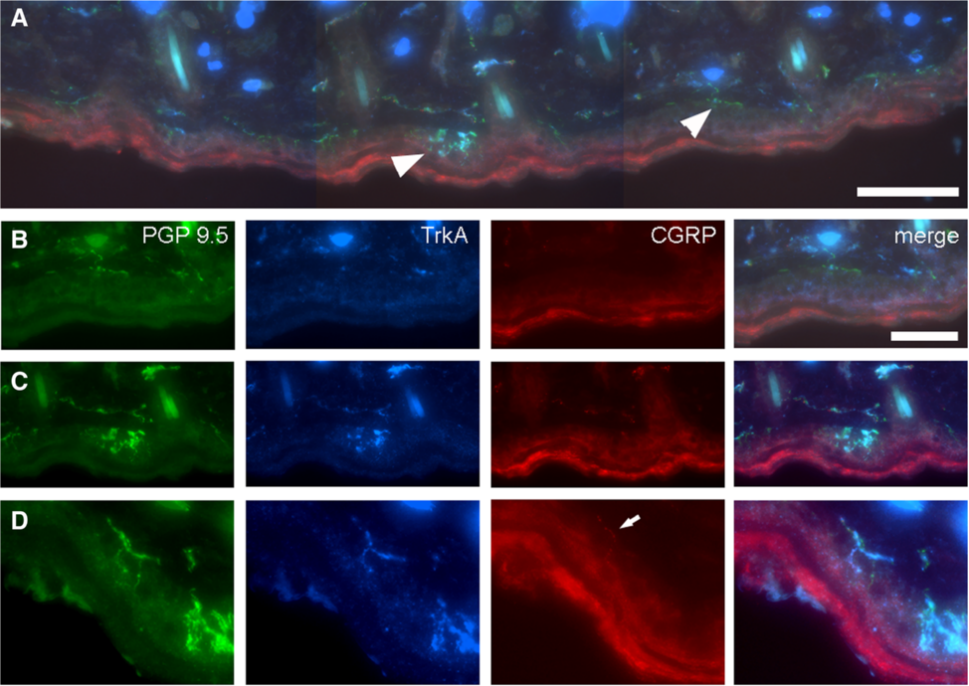
**Examples of intra-epidermal nerve fibres (IENFs) in unoperated (näive) skin.** The low-power image **(A)** shows a strip of control skin that contains several IENFs. Two marked with arrowheads are shown at higher magnification in **B** and **C**. Only one of the IENFS shown at high magnification **(D)** is double labelled for both TrkA (Tropomyosin-related kinase A, blue) and the neuropeptide Calcitonin gene-related peptide (CGRP, red; arrow). Scale bar = 200 μm **(A)**, 10 μm **(B, C)** and 5 μm **(D)**.

In unoperated (naïve) controls most IENFs were varicose and bead-like when labelled with PGP 9.5 or CGRP, however approximately ⅓ formed a dense tangled ‘flower spray’ arrangement that was morphologically similar to the bush/cluster endings described by Fundin *et al*., ([29]; Figure 7) in the intervibrissial fur of the rat. In the present study these ‘flowerspray’ endings were virtually always TrkA-labelled (e.g. Figure 7D). Regardless of terminal morphology, all IENFs extended perpendicular to the basement membrane, and probably originated from tier 4 of the dermal plexus [29] as they could not be followed in continuity to deeper tiers. Occasionally a thicker dermal fibre was in close proximity to a ‘flowerspray’ ending, suggesting that some of this type of IENF ending may arise from tier 2 myelinated fibres [29].

Analysis of PGP 9.5-labelling showed that the IENF density of control (unoperated) skin was 11.6 ± 4.9 fibres/mm (mean ± SD, range 7.3 – 16.7, *n* = 12). TrkA-labelled IENF’s had a density of 4.5 ± 2.0 fibres/mm (mean ± SD, range 1.3 – 7.1, *n* = 12), whereas the density of CGRP-labelled IENF’s was 1.3 ± 0.7 fibres/mm (mean ± SD, range 0.4 – 2.3, *n* = 12). The mean proportion of IENF’s that were TrkA-labelled was 37.8% (range: 17.8 – 52.7, SD 10.2, *n* = 12), and the mean percentage of TrkA-labelled fibres that were also CGRP-positive was 29.4% ± 4.5 (mean ± SD, range: 19.7 – 41.1, *n* = 12); the CGRP-labelled population comprised 10.8% ± 5.4 of the total IENF density (range 5.9 – 23.7, *n* = 12). The mean number of branches on CGRP-labelled fibres was 1.2 ± 0.4 (range: 1 – 2).

**Figure 8:**
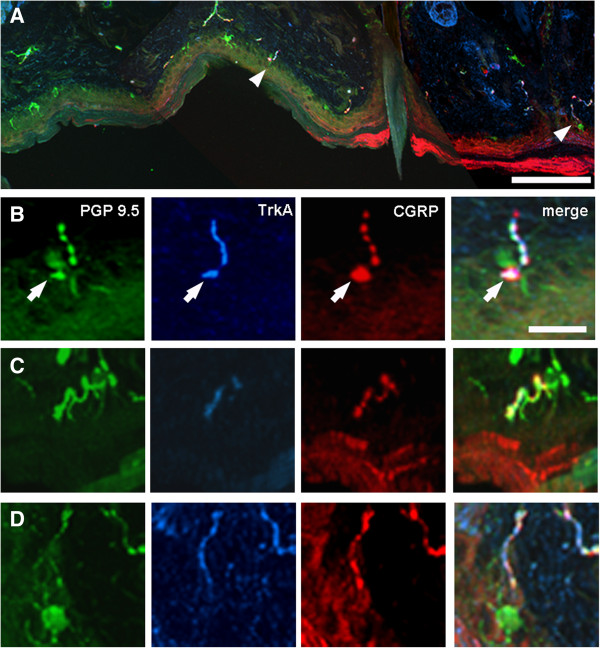
**Examples of intra-epidermal nerve fibres (IENFs) 21 days after chronic constriction injury of the mental nerve.** The low-power image **(A)** shows a strip of partially-denervated skin that contains two IENFs (arrowheads), which are shown at higher magnification in **B** and **D**. Each of the fibres shown at high magnification in **B** – **D** is double-labelled for both TrkA (Tropomyosin-related kinase A, blue) and the neuropeptide Calcitonin gene-related peptide (CGRP, red). In **B** a Merkel disc (arrow) within the epidermis is also CGRP-labelled and a Langerhans cell is labelled with protein gene-product 9.5 (PGP 9.5, green) in **D**. Scale bar = 200 μm **(A)** and 10 μm (**B**; also applies to **C**, **D**).

After CCI, Langerhans cells were more numerous and brightly labelled with PGP 9.5 in just a few samples (Figure 8A, D), but there was no obvious qualitative difference in their appearance compared to Langerhans cells contralaterally. Merkel discs at the base of the epidermis were occasionally CGRP-labelled and were in close association with an IENF (Figure 8B arrow).

**Figure 9:**
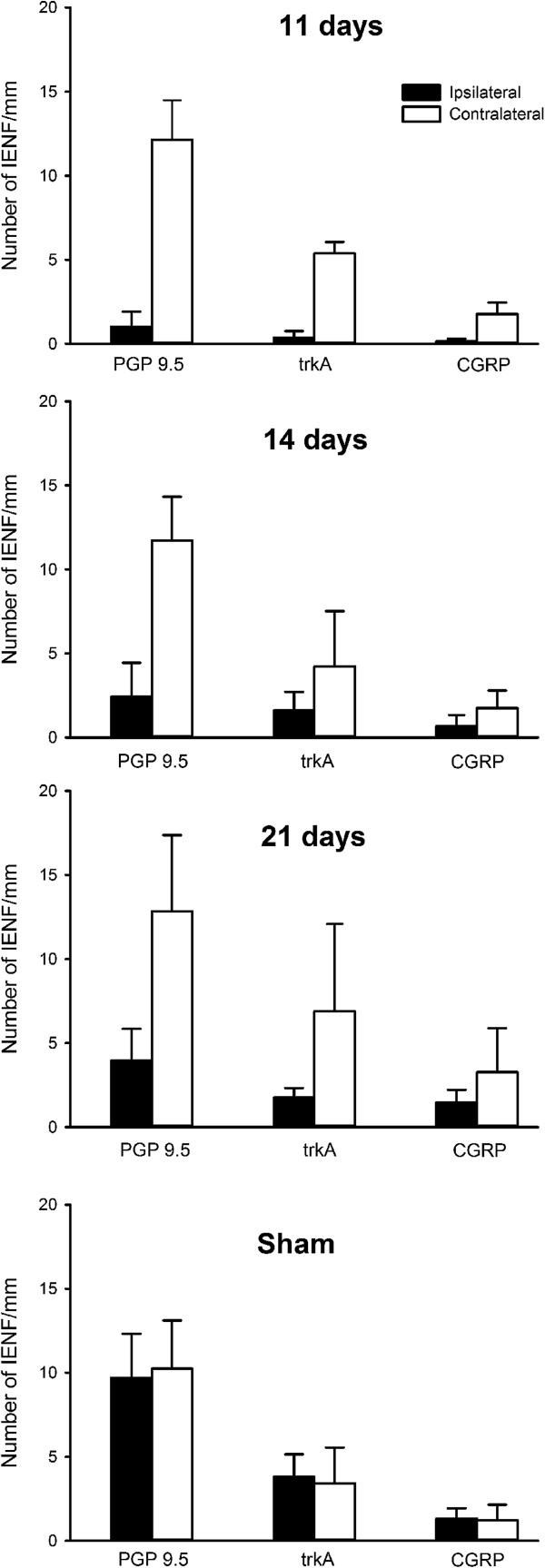
**Intra-epidermal nerve fibre (IENF) density after chronic constriction injury of the mental nerve.** The number of nerve fibres that originated from, or crossed, the basement membrane were counted in 10 μm parasagittal sections that were immunolabelled for protein gene product 9.5 (PGP 9.5), Tropomyosin-related kinase A (TrkA) and Calcitonin gene-related peptide (CGRP).

As expected, the density of IENFs was significantly reduced at all three post-operative time-points studied when compared to the contralateral side (Figures 8 and 9). The extent of denervation was maximal at 11 days post-injury, and all types of IENF were affected regardless of their neurochemical phenotype. From day 11 to day 21 fibre density gradually increased (*P* < 0.008; *ANOVA*), from 1.03 ± 0.37 fibres/mm (mean ± SEM) to 3.95 ± 0.77 fibres/mm, (compare Figure 9, 11 days to Figure 9, 21 days) indicating that some reinnervation occurred over the course of the experiment.

**Figure 10:**
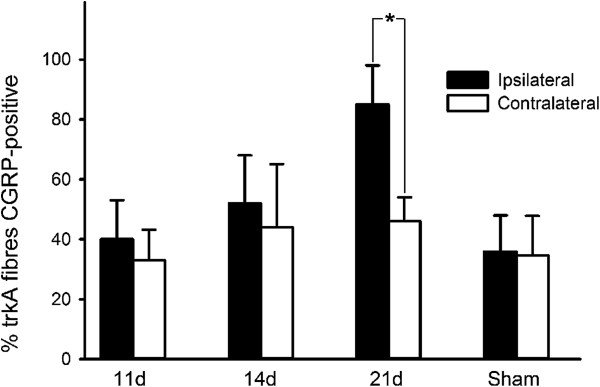
**CGRP labelling of TrkA-positive nerve fibres after CCI.** The proportion of Tropomyosin-related kinase A (TrkA) positive fibres that were double-labelled for Calcitonin gene-related peptide (CGRP) increased significantly on the operated side of CCI animals 21 days after mental nerve injury (*P* < 0.05 Wilcoxon matched pairs test).

After CCI, the proportion of TrkA-positive fibres that were also CGRP-labelled was 40 ± 13% on day 11 and 52 ± 16% on day 14, which was not significantly different to that contralaterally (39 ± 12%, *P* > 0.05, General Linear Model). However, at 21 days post-injury a significantly greater proportion of TrkA-positive fibres were also labelled for CGRP ipsilateral to mental nerve CCI (85 ± 13%) compared to contralaterally (46 ± 8%, *P* < 0.05 Wilcoxon matched pairs test; Figure 10). There was no difference between sides in the proportion of fibres that were TrkA-positive in CCI animals (Figure 10): 11 days, ipsilateral = 54 ± 15.5% (SEM), contralateral = 44 ± 3%, *P* > 0.4, (Wilcoxon matched pairs test); 14 days, ipsilateral = 37 ± 8%, contralateral = 35 ± 11.6%, *P* > 0.8; 21 days, ipsilateral = 50 ± 8.8%, contralateral = 47 ± 9%, *P* > 0.6. This indicates that the TrkA-positive population of IENFs were not preferentially spared following mental nerve CCI.

There was no significant difference in the number of branches on CGRP-labelled fibres in skin ipsilateral to a CCI (1.17 ± 0.11 [mean ± SD], range: 1 – 2) compared to contralateral (1.16 ± 0.19, range: 1 – 3) or unoperated skin (*P* > 0.9,*ANOVA*). This indicates that there was no CGRP-positive IENF sprouting within the epidermis after nerve injury.

## Discussion

The principal finding in the current study is that there is a temporal mismatch between the behavioural signs of neuropathic pain (day 11), skin [NGF] (days 11 & 14) and phenotypic changes in cutaneous IENFs that express the high-affinity NGF receptor TrkA (day 21). To our knowledge this is the first time that the temporal development of neuropathic pain in relation to changes in peripheral NGF concentration and nerve fibre neurochemistry in skin have been studied. There was no significant difference in behavioural response between the injured and non-injured side in sham-operated animals indicating that behavioural changes observed were due to nerve injury and not simply due to localised inflammation caused by the surgery.

### Technical considerations

The present data show that unilateral CCI of the rat mental nerve produced behavioural evidence of neuropathic pain, whereas the PTL model did not. Just one other study has been able to demonstrate neuropathic pain after unilateral mental nerve CCI [30], and in that study only the chemical algogen capsaicin was an effective stimulus, as responses to cutaneous mechanical or heat stimuli were unchanged after injury (cold stimuli were not tried [30]). Bilateral chronic constriction injury of the mental nerves with silk sutures [31-33] produces mechanical allodynia after 2 weeks, so perhaps in the trigeminal system bilateral CCI’s are required to produce the kind of changes in nociceptive behaviours seen after unilateral sciatic nerve injuries [34]. The extent of the denervation of the chin skin after mental nerve CCI (Figure 9) was broadly similar to that seen in the footpad after sciatic nerve CCI [35], indicating that despite using two 6/0 ligatures for mental nerve CCI instead of four 4/0 ligatures that are used for sciatic nerve CCI [34], the proportion of damaged axons is similar in both models. In the PTL model, we were careful not to ligate more than half of the mental nerve, as if too much of the nerve is ligated hypoalgesia or even analgesia can occur [36]. The current results with the PTL showed no hypoalgesia to any of the stimuli used, thus confirming that an appropriate proportion of the mental nerve was ligated. Partial tight ligation of the mental nerve in other studies has also failed to produce neuropathic pain behaviours [30], and this might reflect the different fibre composition of trigeminal *vs*. spinal nerves [37].

ELISA was used to measure NGF concentration because it can measure protein levels in absolute units, and has been used by others to measure NGF levels in skin [38]. The ELISA kit uses both monoclonal and polyclonal antibodies to bind NGF, but the antibodies cannot distinguish between the pro- and mature forms of NGF. Mature NGF is degraded quickly *in vivo*[39], and there is evidence that pro-NGF is up to 10 times more abundant in the nervous system than the mature form [40]. The acid–base treatment that we used increased NGF yield by about 7 fold in pilot studies, and it is thought to promote NGF dissociation from its receptors/binding proteins [41]. We suspect that the acid–base treatment solubilises all the NGF in the tissue, and that the ELISA detects total NGF (both pro- and mature).

### Behavioural signs of neuropathic pain after mental nerve CCI

Only cold hyperalgesia was demonstrable in the mental nerve CCI model, as the changes in behaviour to heat and mechanical stimuli were in the opposite direction (hypoalgesia) and therefore not indicative of neuropathic pain. In our hands, and that of others [30], CCI of the mental nerve seems to be a ‘mild’ model of trigeminal neuropathic pain as there is no behavioural evidence of heat or mechanical hyperalgesia. A previous study of CCI of the rat infraorbital nerve [42] reported that the greater the number of categories of behaviour that are observed in response to a noxious stimulus, the greater the pain severity. In the current study, cold stimuli evoked the greatest number of different behaviours (grooming, withdrawal, wiping) and this could indicate that cold caused the most pain in mental nerve CCI. This might explain why only cold hyperalgesia was detectable in the mental nerve CCI model.

In the CCI model 50% withdrawal thresholds for mechanical stimuli increased over time on the contralateral side, but were stable ipsilaterally, whereas in the sham-operated group thresholds did not change. The method of mechanical testing presented a food reward with the stimulus. It is known that pain responses are suppressed in situations where rats anticipate a reward [43], and because of the overnight fast, the biscuit fed to the rats during mechanical testing will probably have been a motivational reward (“wanting”) [44]. A hedonic (“liking”) reward is also possible due to the sweetness of the biscuit. When both pain (the mechanical stimulus) and reward (biscuit) are presented simultaneously the rats must have made a prioritizing ‘decision’ regarding the value of the reward versus the intensity of the pain. This could explain why mechanical thresholds increased contralaterally over time – the repeated motivation of the reward caused sustained activation of descending control pathways [42] that caused withdrawal thresholds to increase. If this interpretation is correct then the failure of thresholds to rise ipsilaterally could imply that descending inhibitory pathways are less effective after mental nerve CCI. This hypothesis could be tested experimentally if rats could be trained without the need for a food reward for mechanical testing.

Our interpretation fits with Fields [45] framework where pain is described in the context of decision-making under conditions of motivational conflict; he suggests that endogenous opioids suppress responses to stimuli in the presence of competing motivational states such as hunger. Because the mechanical testing took place in the morning after the rats had been starved overnight and the thermal tests were conducted in the afternoon after the animals had been fed, the motivation of hunger was only present in the mechanical test, which explains why only mechanical thresholds increased contralaterally. As noted above, a hedonic motivation for the reward is also possible, and this would involve dopaminergic pathways from the ventral striatum [45]. The pharmacological sensitivity of dopaminergic and opioid descending pathways offers the opportunity to separate motivational from hedonic reward.

As the dominant behavioural sign of neuropathic pain after mental nerve CCI was cold hyperalgesia, a role for the noxious cold-gated ion channel TRPA1 is plausible [46] (but see [47] for contradictory data). TRPA1 expression is upregulated by NGF [48], but when skin [NGF] was increased ipsilaterally on day 14 there was no behavioural evidence of cold hyperalgesia, and conversely when there was behavioural evidence of cold hyperalgesia (day 11) there was no significant difference in skin [NGF] between sides. On the basis of *in-situ* hybridization studies [49] virtually all (97%) of the dorsal root ganglion neurons that express TRPA1 mRNA also co-express mRNA for the noxious heat-gated ion channel TRPV1. Therefore nerve fibres that express TRPA1 ought to co-express TRPV1 and be sensitive to noxious heat, *i.e.* be polymodal nociceptors. As there was no behavioural evidence of heat hyperalgesia in the current study, it would seem unlikely that TRPA1 fibres that co-expressed TRPV1 were involved in cold hyperalgesia. Other potential mechanisms include high-threshold cold receptors [50] as well as cold-induced changes in membrane resistance/resurgent Na^+^ currents [51].

### Effects of nerve injury on skin [NGF] and skin innervation

Eleven days after nerve injury NGF levels were lower bilaterally when compared to sham-operated controls. Fourteen days after chronic constriction of the mental nerve [NGF] was significantly higher ipsilaterally when compared to contralateral skin, although there was no difference between CCI and sham-operated animals. At this time point, the maximum difference in NGF concentration between sides was 42 pg/mg for skin samples that weighed on average 65 mg. This gives a rough estimate of an increase of 3 ng of NGF after mental nerve CCI (42 pg/mg × 65 mg = 2730 pg), which is 1000 fold smaller than exogenous levels that have biological activity in nerve sprouting and pain studies [14,18,52,53]. As described previously [54] and replicated here, skin [NGF] changed bilaterally after a unilateral nerve injury. As there was no change in NGF mRNA levels in the skin territory of the injured nerve [54] the contralateral [NGF] increase may be due to NGF diffusion secondary to reduced axonal transport post nerve injury.

Approximately 40% of IENFs in the normally innervated chin skin were immunoreactive for TrkA, which is similar to the proportion of TrkA-positive cutaneous hindpaw neurons in the DRG [6]. 30 - 40% of the TrkA-labelled fibres were also CGRP-positive, and in the epidermis these are likely to represent the terminals of cutaneous nociceptors [55]. The observation that not all of the TrkA-labelled IENFs were CGRP-positive [19,56] is likely due to the fact that more CGRP-positive fibres are confined to the dermis, and do not branch into the epidermis [32,33].

### Neuropathic pain, [NGF] and IENF neurochemistry

Eleven days after CCI there was behavioural evidence of cold hyperalgesia. Over the next 10 days [NGF] increased threefold bilaterally but there was no evidence of neuropathic pain. Thus NGF and cold hyperalgesia do not seem to be linked in the trigeminal system, although there does seem to be a causal link in spinal nerves, [57,58]. This indicates a difference in the aetiology of neuropathic pain between spinal and trigeminal nerves.

## Conclusions

We had previously proposed [59] that in partial nerve injuries an excess of NGF peripherally ‘over-trophed’ the remaining peripheral sensory nerve fibres, producing a ‘pro-nociceptive’ phenotype, therefore being a candidate peripheral mechanism for neuropathic pain. The current experiments do not support this hypothesis at least in the trigeminal system, as when skin [NGF] was significantly increased there was no behavioural evidence of neuropathic pain. The phenotypic changes in IENFs could be due to increased [NGF] in the skin, but they do not appear to be sufficient on their own to cause neuropathic pain.

## Methods

### Animals

Experiments were performed on 76 male Sprague–Dawley rats (Charles River, UK; 150 – 200 g). The experiments were approved by the Ethical Review Panel at Sheffield University and licensed under the UK Animals (Scientific Procedures) Act 1986. The animals were divided into 4 groups: (1) Animals that had a chronic constriction injury of a mental nerve (*n* = 28); (2) Animals that had a partial tight ligation of a mental nerve (*n* = 24); (3) Sham-operated controls (*n* = 18) and (4) Näive (unoperated) controls (*n* = 6).

### Neuropathic pain models

Rats were anaesthetized with Isoflurane (4% induction, 2% maintenance) and the left mental nerve exposed surgically via an incision along the lower border of the mandible. A short segment of nerve, just distal to the mental foramen where it consisted of just a single nerve trunk, was dissected free. In 28 rats the nerve was loosely ligated with two 6/0 chromic gut sutures tied at 1 mm intervals. The sutures slightly indented the epineurium, which is in keeping with the original description of the sciatic nerve chronic constriction injury (CCI) [34]. It was not possible to use the standard 4 ligatures that sciatic nerve CCI uses because there is only a short length of mental nerve available after it exits the mental foramen before the nerve separates into smaller branches. In 24 animals, one-third to one-half of the nerve was tightly ligated with a single 6/0 silk suture in approximately the same place as the ligatures in the CCI model. This partial tight ligation model (PTL, based on [36]) was trialled as a more reproducible method of producing neuropathic pain, as the degree of tension in the ligatures in the CCI model cannot be absolutely standardised. Sham-operated animals (*n* = 18) had the nerve surgically exposed but no further manipulation. The nerve injury and sham-operations were always performed unilaterally as there is no significant transmedian innervation of the chin skin from contralateral mental nerve afferents [60]. The incision was closed in layers and a single dose of amipicillin (15 mg; Duphacillin, Fort Dodge Animal Health, UK) given intramuscularly in the right thigh. The local anaesthetic lignocaine (0.5 ml of a 2% solution) was injected into the surgical site for post-operative analgesia. The animals recovered under observation.

### Behavioural testing

Rats were maintained on a 12 hour light/dark cycle with food and water provided *ad libitum* and housed 4 per cage. Behavioural testing to determine baseline nociceptive sensitivity was performed 6, 3, and 1 day pre-operatively and then 1, 4, 6, 8, 11, 14, 18 and 21 days post-operatively. Responses evoked by mildly noxious mechanical, cold and heat stimuli applied to the skin of the lower lip and chin were recorded. For mechanical sensitivity testing a series of calibrated von Frey filaments (Stoelting, Wood Dale IL, USA) was used. Cold sensitivity was tested using topical ethyl chloride applied on a cotton bud and heat sensitivity was measured using radiant heat (IITC, Woodland Hills CA, USA).

Testing the mechanical sensitivity of the mental nerve territory using von Frey filaments was initially fruitless because the animals were too inquisitive to obtain meaningful data from. Therefore the rats were trained to accept a food reward (McVities Digestive, United Biscuits, Hayes, UK) from the fingers of the experimenter whilst rearing on their hind legs. This posture exposed the ventral lower lip and chin to the experimenter, and the rats were trained to stand like this, continually eating, so that mechanical stimuli could be applied to the mental nerve innervation territory. Rats were trained over a 12 day period prior to behavioural testing, and only animals that displayed normal exploratory or grooming behaviour during handling after this 12 day period were used further. Rats that could not be trained to take the food reward were not studied further. To expedite the rats taking the food reward, von Frey testing was conducted in the morning after an overnight fast. Rats were provided with standard diet afterwards. The contralateral, uninjured (right) side was used for control measurements. The experimenter who performed the behavioural testing was blinded to the experimental grouping.

Testing of mechanical sensitivity occurred in the home cage after an overnight fast. Mechanical testing was omitted on the first post-operative day so the animals were able to recover from the preparatory surgery with minimal interference. The ‘up-down’ method using a series of graded von Frey monofilaments (1.6, 2, 4, 8, 15, 26, 33, 40 g) was used to calculate 50% withdrawal thresholds [61]. Briefly, testing began with the 8 g filament, which was applied perpendicular to the skin surface until it just bent. A flinch or fast withdrawal was interpreted as a positive (aversive) response. Aversive responses were usually accompanied by a small and brief withdrawal away from the stimulus but occasionally the withdrawal was more prolonged or accompanied by an attack response (biting the filament). Rarely, a brief grooming of the stimulated area was elicited. Withdrawals that lacked rapidity and were unflinching were usually accompanied by the rat turning its attention toward the source of the stimulus (orienting head towards stimulus, sniffing) and this was interpreted as inquisitive behaviour, indicative of non-aversive perception of the stimulus. The stimulus was repeated if there was any doubt that the response was aversive or not. A positive response was followed by applying the next lowest bending force filament, a negative response was followed with the next highest bending force filament. A series of 6 stimuli that oscillated around threshold were sufficient to calculate the 50% withdrawal threshold [61]. In the event that none of the stimuli evoked withdrawal behaviours, the 50% threshold was assigned to 40 g.

Testing cold and heat sensitivity required the animals to be soporific, so data were recorded in the afternoon after the animals had eaten. Rats were placed in a mesh floored cage and tests were undertaken when they became stationary, ceased exploratory behaviour and their eyes were open but narrowed. To perform the tests the animals had to rest on all fours so that the lower lip and chin were unobstructed and parallel to the cage floor.

Ethyl chloride, which cools by evaporation, was sprayed onto a cotton bud that was applied from below through the mesh floor to the left or right side of the chin. The skin was briefly touched with the ethyl chloride soaked cotton bud, which was cold enough to cause frosting of the hairs of the rats’ skin. The duration of directed bilateral grooming evoked within one minute of this stimulus was cumulatively recorded with a stopwatch [62]. If non-directed grooming as part of a general grooming sequence occurred during this time then any subsequent directed grooming was not recorded. A minimum interval of 5 minutes was allowed between stimuli. Stimuli were applied alternately to the left and right side, with the side of the initial testing determined at random and average responses to 3 stimuli per side were recorded. An aversive effect of the sound of the ethyl chloride spray was prevented by applying a neutral temperature stimulus (dry cotton bud) after sounding the spray alternately between applications of the cold stimulus throughout the course of the experiment. The odour of the ethyl chloride was also controlled for by intermittently presenting a cotton bud soaked in ethyl chloride to the rat’s nose while the lower lip and chin were touched by a dry cotton bud.

Heat sensitivity was measured by recording the latency of withdrawal from a radiant heat source [63] that was focussed on either the right or left side of the chin. The intensity of the source was adjusted to evoke withdrawals with latencies of 7 - 12 s in naïve animals. An average from 3 withdrawal latency measurements for each side was calculated with a minimum interval of 5 minutes between each stimulus, and stimuli were applied alternately to the left and right side, with the side of the initial testing determined at random. To prevent thermal injury to the skin the radiant heat stimulus automatically cut-off if the animal did not withdraw after 20 s.

### Measurement of skin [NGF]

**Figure 11:**
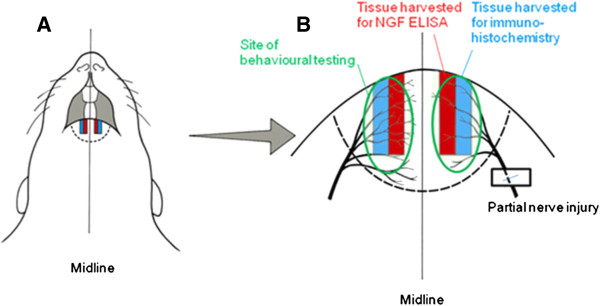
**Methods.** Illustration of the ventral aspect of the rat facial soft tissues **(A)** and a magnified view of the lower lip and skin innervation territory of the mental nerve **(B)**. A partial injury of the left mental nerve was performed either by loosely ligating it with two 6/0 chromic gut sutures or by tightly ligating ⅓ to ½ of its diameter with a single 6/0 silk suture. The areas stimulated for behavioural testing are outlined and the tissue harvested for NGF quantification and innervation density measurement are also indicated.

Tissue was harvested from the lower lip for measurement of [NGF] using a sandwich enzyme-linked immunosorbent assay (ELISA). Under terminal barbiturate anaesthesia (Sodium pentobarbital, 200 mg/kg I.P.), a 2 mm wide strip of tissue 1 mm from the midline of the lower lip was excised (Figure 11) and snap frozen in liquid nitrogen. The tissue one millimetre either side of the midline was not used as after nerve injury there is collateral sprouting from the contralateral mental nerve, and individual fibres can cross the midline by up to 1 mm [60]. Tissue was stored at -80°C before processing. To enrich the study, tissue from rats that showed the largest differences between cold-evoked grooming behaviour on post-operative day 11 were selected for assay (*n* = 6 per group).

Samples were weighed, minced and homogenised (2 minutes at 10,000 rpm; Micra-D8, ART-Labortechnik, Müllheim, Germany) in 1 ml ice-cold extraction buffer that contained 100 mM Tris HCl, 1 M NaCl, 4 Mm EDTA, 2% Triton X-100 and a cocktail of protease inhibitors (cOmplete, Mini, EDTA-free, Roche, Burgess Hill, UK) dissolved in distilled water. To solubilise and isolate NGF from binding proteins (including TrkA) an acid–base treatment was used [64]. Omitting this step measures only free mature NGF (manufacturers specification). In pilot studies, only 9.0 ± 0.3 pg/mg of NGF was detected when the acid–base treatment of lip skin homogenates was omitted, and this increased to 67.4 ± 3.7 pg/mg (mean ± 1 SD, *n* = 3 per group) with acid–base treatment, suggesting that the treatment facilitated the detection of all forms of NGF. Samples were alkalized to pH 11 with 4 M NaOH and centrifuged at -4°C for 15 minutes at 10,000 rpm. The supernatant was separated from the pellet and acidified to pH 3 with glacial acetic acid and centrifuged at -4°C for 30 minutes at 10,000 rpm. The supernatant was again separated from the pellet and then neutralized with 4 M NaOH and cold centrifuged at -4°C for a further 15 minutes at 10,000 rpm. The supernatant was then aliquoted and stored at -80°C.

The supernatant was assayed by colourimetry using a commercial kit (NGF E_max_®ImmunoAssay System, Promega, UK) and the results read with an absorbance reader (Infinite M200, Tecan, Switzerland) at 450 nm. NGF content was quantified against a standard curve with known amounts of protein (3.9 – 250 pg/ml). Optical densities were adjusted by subtracting background, correcting for wet weight and dilution. Measurements were performed in duplicate and expressed as pg per mg of wet tissue weight. The lower limit of sensitivity for the assay was 7.8 pg/mg.

### Density and phenotype of intra-epidermal nerve fibres

Samples of lip skin that were processed to investigate intra-epidermal nerve fibre density and phenotype were obtained immediately lateral to the samples obtained for ELISA (Figure 11), after the animals had been perfused with fixatives. Reduced intra-epidermal nerve fibre density is known to be associated with neuropathic pain in humans [65], but other nerve fibres in the dermis are also involved in nociceptive processing [66] Under terminal barbiturate anaesthesia, rats were perfused transcardially with 200 ml of phosphate buffered saline (PBS) followed by 200 ml of freshly depolymerized 4% paraformaldehyde. Equivalent portions of the lower lip and skin of the chin were excised from the injured and non-injured sides (see Figure 11), and were post-fixed in 4% paraformaldehyde for 4 hours. The tissue was then washed in phosphate buffered saline (PBS) and cyroprotected in 30% sucrose solution overnight. The tissue was embedded in Tissue Tek OCT (Sakura, Thatcham, UK) and 10 μm para-sagittal sections cut on a cryostat that were mounted on poly-D-lysine coated slides. Sections were collected as 20 sets of 4 slides with 3 sections per slide. Thus adjacent sections on each slide were separated by 200 μm and each set contained tissue representing the entire medio-lateral extent of the sample. The sections were air dried for 60 minutes before being stored at -80°C.

Indirect immunofluoresence was used to triple label sections with primary antibodies raised against protein gene-product 9.5 (PGP 9.5, a general neuronal marker), Calcitonin gene-related peptide (CGRP, labels ≈ 40% of cutaneous nociceptors and a few G-hair mechanoreceptors, [55]) and TrkA (the high affinity NGF receptor) to identify putative NGF-dependent nociceptors. Primary antisera were diluted in PBS with 5% normal donkey serum and incubated at 4°C overnight. The primary antibodies used were anti-PGP 9.5 raised in rabbit (UltraClone, Cambridge, UK diluted 1:1000); anti-CGRP raised in guinea pig (Bachem, St. Helens, UK diluted 1:6000) and anti-TrkA raised in goat (R&D Systems, Abingdon, UK diluted 1:5000). The intensity of labelling with the TrkA antibody was weak and therefore signal amplification with tryamide conjugated to Coumarin (PerkinElmer Inc., Cambridge, UK) was used. Endogenous peroxidase activity was quenched after incubation in the primary antibody by washing the slides with PBS 3 × 6 minutes and then immersion in 0.3% H_2_O_2_ and 0.1% sodium azide in PBS for 15 minutes. Sections were incubated in a donkey anti-goat secondary antibody that was conjugated to biotin for 90 minutes (Stratech, diluted 1:5000). Incubation in horseradish peroxidase conjugated to streptavidin for 5 minutes was used to deposit the coumarin-tyramide onto the tissue. Sections were then incubated in primary antisera against PGP 9.5 and CGRP as described. After washing the slides in PBS for 2 × 10 minutes they were incubated for 90 minutes at room temperature in secondary antibodies that were conjugated to fluorophores and diluted in PBS with 1.5% normal donkey serum. PGP 9.5 labelling was revealed with a donkey anti-rabbit antibody labelled with fluorescein isothiocyanate (FITC; Stratech, Newmarket, UK, diluted 1:200); CGRP labelling was revealed with a donkey anti-guinea pig secondary antibody that was conjugated to indocarboyanine (Cy3; Stratech, diluted 1:500). Finally, sections were washed (2 × 10 minutes in PBS), mounted in Vectorshield (Vector Laboratories, Peterborough, UK) and coverslipped.

Negative controls were performed by omitting the primary antibodies. Positive controls were undertaken by preabsorbing the primary antibodies with an excess of their immunogenic proteins (10 nmol/ml) for the TrkA and CGRP antibodies. The preabsortion proteins for TrkA and CGRP were reconstituted with sterile PBS and incubated for 1 hour at room temperature with their antibodies. The PGP 9.5 preabsortion control came pre-diluted from the manufacturer.

Sections were viewed with an Axioplan 2 microscope (Zeiss, UK) and a dry ×40 objective lens. Digital images were captured (Qimaging Retiga 1300R) and stored as TIFF files. The total length of epidermis in each section was measured in 10 consecutive fields of view using software (ImagePro® Plus Media Cybernetics Inc, Rockville MD, USA), starting at the junction of the vermillion border and hairy skin. Intra-epidermal nerve fibres (IENF) were quantified using the European Federation of Neurological Societies guidelines [65]. The guidelines specify that only fibres that either cross or originate at the basement membrane are counted. Branching within the epidermis is not counted. Thus the method excludes increased fibre branching as a cause of increased immunolabelling, but to investigate this explicitly, the number of branches for every CGRP immunolabelled fibre was recorded for skin samples obtained 21 days after nerve injury, when the post-injury innervation density was maximal. The method of quantification was repeatable to within 95% of 2 SD. Only structures that could be unequivocally identified as nerve fibres, by the presence of varicosities, were counted to avoid counting Langerhans cell dendrites. Langerhans cells are PGP 9.5 immunolabelled and appear more numerous and more intensely labelled in denervated skin [67], but they were easily recognised by the presence of a cell body with smooth dendrites extending from it (Figure 8B).

The average PGP 9.5-positive fibre count per millimetre of basement membrane was calculated for each section. The proportion of fibres labelled for both TrkA and CGRP as well as differences in the mean number of IENF labelled with antibodies against PGP, CGRP and TrkA were also determined.

### Statistical analysis

Behavioural responses over time were analyzed using 2-factor repeated measures Analysis of Variance (RM ANOVA) with a confidence level of 0.05. If a significant difference was found, *post-hoc* Tukey’s tests were performed with *P* < 0.05 considered significant. For 50% withdrawal thresholds to mechanical stimuli, difference scores were obtained by subtracting thresholds of the un-operated right side from those of the operated left side. Difference scores were analyzed using the General Linear Model ANOVA for non-parametric data.

ANOVA was used to compare changes in skin [NGF] over time. A *post-hoc* independent *t*-test was used to compare skin [NGF] between different time-points with a Bonferroni correction for *n* = 4 groups applied (see Results), so that a *P* value < 0.0125 was needed for significance.

Statistical comparisons of fibre counts were made between the nerve-injured and sham-operated animals over time were made using ANOVA with a Bonferroni correction for *n* = 3, so that a *P* value < 0.017 was considered significant. The Wilcoxon matched pairs test was used to compare proportions of fibres in different groups that were immuno-positive for TrkA and CGRP, with *P* < 0.05 being significant.

## Abbreviations

CCI: Chronic constriction injury; CGRP: Calcitonin gene-related peptide; ELISA: Enzyme-linked immunosorbant assay; IENF: Intra-epidermal nerve fibre; NGF: Nerve growth factor; PGP 9.5: Protein gene-product 9.5; PTL: Partial tight ligation; TrkA: Tropomyosin-related kinase A.

## Competing interests

The authors declare that they have no competing interests.

## Authors’ contributions

LE performed the behavioural testing, ELISA and immunocytochemistry. AL conceived the study and participated in its design and coordination. FB participated in study design and immunocytochemistry. SW participated in the ELISA studies. PR performed all surgery and participated in study design. DA performed the statistical analyses, participated in study design and drafted the manuscript. All authors read and approved the final manuscript.
